# The Non-Steroidal Mineralocorticoid Receptor Antagonist KBP-5074 Limits Albuminuria and has Improved Therapeutic Index Compared With Eplerenone in a Rat Model With Mineralocorticoid-Induced Renal Injury

**DOI:** 10.3389/fphar.2021.604928

**Published:** 2021-06-24

**Authors:** Frédéric Jaisser, Xiaojuan Tan, Shuangshuang Chi, Jinrong Liu, Ping Wang, Mark Bush, Vincent Benn, Y. Fred Yang, Jay Zhang

**Affiliations:** ^1^ INSERM UMRS1138, Sorbonne Université, Université de Paris, Centre de Recherche des Cordeliers, Paris, France; ^2^ KBP BioSciences Co., Ltd., Shandong, China; ^3^ KBP BioSciences USA Inc., Princeton, NJ, United States; ^4^ Nuventra Inc., Durham, NC, United States

**Keywords:** mineralocorticoid receptor, urinary albumin to creatinine ratio, UACR, hyperkalemia, therapeutic index, nephropathy, non-steroidal

## Abstract

The therapeutic indices (TIs) and efficacy of the non-steroidal mineralocorticoid receptor antagonist (MRA) KBP-5074 and steroidal MRA eplerenone were evaluated in a uninephrectomized Sprague Dawley rat model of aldosterone-mediated renal disease. In two parallel studies, rats were placed on a high-salt diet and received aldosterone by osmotic mini-pump infusion over the course of 27 days. The urinary albumin-to-creatinine ratio (UACR) was evaluated after 7, 14, and 26 days of treatment. Serum K^+^ was evaluated after 14 and 27 days of treatment. Urinary Na^+^, urinary K^+^, and urinary Na^+^/K^+^ ratio were evaluated after 7, 14, and 26 days of treatment. The TI was calculated for each drug as the ratio of the concentration of drug producing 50% of maximum effect (EC_50_) for increasing serum K^+^ to the EC_50_ for lowering UACR. The TIs were 24.5 for KBP-5074 and 0.620 for eplerenone, resulting in a 39-fold improved TI for KBP-5074 compared with eplerenone. Aldosterone treatment increased UACR, decreased serum K^+^, and decreased urinary Na^+^ relative to sham-operated controls that did not receive aldosterone infusion in both studies, validating the aldosterone/salt renal injury model. KBP-5074 prevented the increase in UACR at 0.5, 1.5, and 5 mg/kg BID while eplerenone did so only at the two highest doses of 50 and 450 mg/kg BID. Both KBP-5074 and eplerenone blunted the reduction in serum K^+^ seen in the aldosterone treatment group, with significant increases in serum K^+^ at the high doses only (5 mg/kg and 450 mg/kg BID, respectively). Additionally, the urinary Na^+^ and Na^+^/K^+^ ratio significantly increased at the middle and high doses of KBP-5074, but only at the highest dose of eplerenone. These results showed increased TI and efficacy for KBP-5074 compared with eplerenone over a wider therapeutic window.

## Introduction

Chronic kidney disease (CKD) is major contributor to the global burden of disease, increasing the risks of morbidity and mortality associated with cardiovascular diseases, diabetes, hypertension, human immunodeficiency virus (HIV) infection, and malaria, among others. Globally, an estimated 1.2 million people died from kidney failure in 2015, an increase of 32% from 2005 (Luyckx et al., 2018). Both blood pressure (BP) control and a reduction in proteinuria have been associated with slowing glomerular filtration rate (GFR) decline and, therefore, CKD progression (Lea et al., 2005; Anderson et al., 2015). Renin–angiotensin–aldosterone system (RAAS) inhibitors, such as angiotensin-converting enzyme inhibitors (ACEI) and angiotensin-II receptor blockers (ARB), have historically been the main tools available to clinicians to slow GFR decline but, ultimately, do not stop progression, with a large number of patients developing macroalbuminuria and end-stage kidney disease, possibly due to increased levels of angiotensin II and aldosterone or aldosterone alone (Sarafidis and Ruilope, 2012; Vejakama et al., 2017).

A large amount of evidence, both experimental and clinical, has shown that aldosterone mediates vascular injury and kidney damage through its role in BP control, RAAS-induced vascular injury and nephropathy, and renal remodeling independently of other components of the RAAS (Hollenberg, 2004). Aldosterone binds to the mineralocorticoid receptor (MR) in the epithelium of the distal nephron, playing a part in sodium reabsorption and renal potassium regulation and, therefore, volume regulation and BP control (Jaisser and Farman, 2016). Additionally, expression of the MR has been found in non-classic kidney tissues, such as the vascular endothelium and smooth muscle cells, as well as inflammatory cells, podocytes, and fibroblasts, which may be pathophysiological (Jaisser and Farman, 2016).

Based on these findings, MR antagonists (MRAs) have emerged as important pharmacological treatments for patients with CKD. Numerous studies have found that MRA therapy significantly decreases the urine albumin-to-creatinine ratio (UACR), showing efficacy in the treatment of patients with CKD (Barrera-Chimal et al., 2019). Two recent meta-analysis reviews of randomized controlled trials that evaluated the effects of combination treatment of MRAs and ACEI/ARB concluded that MRAs either alone or in combination with ACEI/ARB resulted in significant antiproteinuric effects compared with ACEI/ARB therapy alone (Alexandrou et al., 2019; Zuo and Xu, 2019). However, patients with CKD, heart failure (HF), or cardiometabolic syndrome, or those taking ACEI/ARB therapy are at risk of developing hyperkalemia (Palmer, 2004). Serum K^+^ levels both above and below the normal range have been shown to be consistently associated with adverse clinical outcomes across a broad range of patient populations, but particularly in patients with CKD or HF (Palaka et al., 2020). MRAs as monotherapy are associated with a risk of hyperkalemia in patients with HF or CKD. When MRAs are combined with ACEI/ARB therapy, the risk of hyperkalemia is higher vs. ACEI/ARB alone (Trevisan et al., 2018; Zuo and Xu, 2019). Episodes of hyperkalemia often result in discontinuation of MRA therapy (Trevisan et al., 2018; Zuo and Xu, 2019). Therefore, the greater benefit of MRAs on UACR lowering vs. ACEI/ARB monotherapy may be negated by an increased risk of hyperkalemia.

More recently, non-steroidal MRAs have been proposed as superior alternatives to steroidal MRAs. Non-steroidal MRAs have been shown to have both BP lowering and cardiorenal protective effects, similar to those of ACEI/ARB therapies, matched with strong MR inhibitory action, high selectivity, and a possible reduced risk of hyperkalemia compared with steroidal MRAs (Sueta et al., 2020). Spironolactone and eplerenone are currently the only steroidal MRAs available. Although several non-steroidal MRAs are being developed and investigated, only esaxerenone, approved in Japan in 2019, is available on the market. The availability of an MRA that effectively decreases UACR without increasing the risk of hyperkalemia would be of tremendous clinical use in the treatment of patients with HF and CKD, among others.

The aim of these parallel studies was to compare the therapeutic indices (TIs) and efficacy of eplerenone and KBP-5074, a novel, highly selective non-steroidal MRA, in a uninephrectomized Sprague Dawley (SD) rat model of aldosterone-induced renal damage. The TIs were calculated as the ratio of the concentration of drug producing 50% of maximum effect (EC_50_) for increasing serum K^+^ to the EC_50_ of lowering UACR. Additionally, we measured the urinary Na^+^/K^+^ ratio to evaluate the natriuretic impact of eplerenone and KBP-5074.

## Materials and Methods

All procedures were conducted in accordance with Institutional Animal Care and Use Committee (IACUC) guidelines in compliance with the Animal Welfare Act, the Guide for the Care and Use of Laboratory Animals, and the Office of Laboratory Animal Welfare, as well as WuXi standard operating procedure regulations.

### Compounds: KBP-5074 and Eplerenone

KBP-5074 was provided by KBP BioSciences Co., Ltd. as a solid dispersion with a purity of 10.6%. The drug was stored at −20°C until use and dissolved in sterile double-distilled water for dosing. Eplerenone was provided by Shanghai Tebo Chemical Technology Co., Ltd. as a powder with a purity of 98%, was stored at room temperature until use per manufacturer’s recommendations, and was prepared for dosing in 0.5% methyl cellulose and 0.1% polysorbate 80 in sterile double-distilled water.

Eplerenone doses used in the study were chosen to match those from a published study comparing eplerenone to the non-steroidal MRA PF-0388285 (Orena et al., 2013) to allow comparison of results. KBP-5074 doses were selected based on previous experiences in SHR-SP and Dahl Salt-Sensitive rat models.

### Animals and Study Design

#### Effect of Administration of KBP-5074 and Eplerenone on Aldosterone-Mediated Renal Injury in the Uninephrectomized SD Rat Model

Two parallel studies, one for each drug, were performed using the same study design and control groups for each, as described below.

##### Study A

Seventy-two male SD rats (SLAC, Shanghai, China), 7–8 weeks of age, were housed singly in a controlled environment and provided a standard diet and water *ad libitum*. After an acclimation period of 5–10 days, pre-surgery baseline 24-h urine samples were collected and used for calculation of UACR. Pre-surgery baseline blood was collected via tail vein and analyzed for serum K^+^. The rats then underwent left nephrectomy under pentobarbital Na anesthesia. After a second acclimation period of 5 days, post-surgery baseline 24-h urine and blood samples were collected and analyzed to determine the UACR, the Na^+^/K^+^ ratio, and serum K^+^. On Day 1 of the study, rats were randomized into five groups based on UACR and serum K^+^ values. All rats (*n* = 11 for group 1, *n* = 13 per group for 2, 3, 4, and 5) then underwent subcutaneous implantation of Alzet mini-pumps (Alzet, Cupertino, CA, United States) under isoflurane anesthesia. To establish the model of aldosterone-induced renal damage, mini-pumps delivered either 2.5 μl/h 0.01% DMSO (group 1, Vehicle Sham) or 2.5 μl/h aldosterone 0.3 mg/ml in 0.01% DMSO (groups 2–5) throughout the 27-day duration of the study. From Day 1, groups 3–5 also received eplerenone at doses of 15 mg/kg (group 3, low-dose group), 50 mg/kg (group 4, mid-dose group), and 450 mg/kg BID (group 5, high-dose group) by oral gavage at 9:00 am and 7:00 pm for 26 days. Rats were switched to a 6% high-salt diet (Teklad TD 90230) and water containing 0.3% KCl *ad libitum* immediately following the mini-pump implantation.

##### Study B

Seventy-six male SD rats were obtained for Study B, which was performed using the same protocol used for Study A, with one difference: KBP-5074 was administered instead of eplerenone at doses of 0.5 mg/kg (group 3, low-dose group), 1.5 mg/kg (group 4, mid-dose group), and 5 mg/kg BID (group 5, high-dose group) by oral gavage.

#### Physical Condition, Body Weight, and Food and Water Intake

Physical condition was assessed by standard animal health evaluation. Body weight was measured and recorded upon receipt, at pre-surgery baseline, post-surgery study baseline, and before every administration. Food and water intake were measured and recorded from Day 1 until the end of the study.

#### Urine Collection

Twenty-four-hour urine samples were collected and urine volume recorded at the pre-operative baseline, post-surgery study baseline, and Days 7, 14, and 25 of the respective studies and used for measurement of UACR, Na^+^, K^+^, and to calculate the Na^+^/K^+^ ratio.

#### Plasma/Serum Collection

Blood was collected at pre-surgery baseline, post-surgery study baseline, Day 14 (7 h post first dose), and Day 27 at necropsy by tail bleed for measurement of serum K+.

Blood was collected at pre-surgery baseline, post-surgery study baseline, Day 1 (1, 2, 4, and 7 h post first dose), Day 14 (7 h post first dose), Day 26 (1, 2, 4, and 7 h post first dose [plus 10 h post first dose for KBP-5074]), and Day 27 at necropsy by tail bleed (eplerenone only) for measurement of plasma drug levels.

#### Tissue Collection

Rats were euthanized on Day 27 with CO_2_. Kidneys were removed, longitudinal sections were fixed in 10% formalin, and the tissue was embedded in paraffin.

#### Biomarker Measurements

Serum K^+^, urinary creatinine, and urinary Na^+^, K^+^, and Na^+^/K^+^ ratio were measured using a clinical analyzer (Hitachi 7180, Japan). Urinary albumin was measured using an ELISA kit (Abcam, United Kingdom). UACR was calculated as the ratio of urinary albumin concentration (µg/ml) to urinary creatinine concentration (µmol/L).

#### Compound Exposure

Plasma concentrations of KBP-5074 and eplerenone were determined using liquid chromatography-tandem mass spectrometry (LC-MS/MS). Chromatographic separations were achieved with a gradient program consisting of mobile phase A (0.1% [volume per volume; V/V] formic acid in water) and mobile phase B (0.1% [V/V] formic acid in acetonitrile) with a flow rate of 0.6 ml/min. ACQUITY UPLC BEH C8 column (1.7 μm, 2.1 × 50 mm) was used (Waters, Milford, MA, United States). For KBP-5074, the lower limit of quantitation (LLOQ) was 0.1 ng/ml and calibration standard range was 0.1–500 ng/ml; the column temperature was maintained at 60°C; the injection volume was 4 μl; the gradient was 20% mobile phase B maintained for 0.2 min, then increased to 95% over 1.0 min and kept there until 1.3 min, then returned to 20% at 1.31 min, run time 1.5 min.

For eplerenone, the LLOQ was 2 ng/ml and calibration standard range was 2–3,000 ng/ml; the column temperature was maintained at 45°C; the injection volume was 3 μl; the gradient was 5% mobile phase B maintained for 0.3 min, then increased to 45% over 0.9 min, to 95% over 1.2 min, then returned to 5% at 1.51 min.

#### Pharmacokinetic (PK) Modeling for Eplerenone and KBP-5074

Plasma levels of KBP-5074 and eplerenone from Day 1 and Day 26 collected at 1, 2, 4, and 7 h post first dose were used to determine maximum plasma drug concentration (C_max_) and area under the concentration time–curve from zero to last measurement (AUC_0-last_). PK modeling analysis for eplerenone and KBP-5074 was performed by FMD K&L (Fort Washington, PA, United States) and used data for groups 2–5 of studies A (eplerenone) and B (KBP-5074). For PK modeling for KBP-5074, pre-dose data for serum K^+^ and UACR were not used. Day 7 data were only available for UACR and not K^+^. A turnover model was selected for analysis of delayed effects and fitted using post-hoc PK parameters. Stochastic approximation expectation maximization (SAEM) was used with the R package nlmixr 1.1.1.1. The EC_50_ was estimated for serum K^+^ and UACR for both KBP-5074 and eplerenone. The TI was then calculated for both KBP-5074 and eplerenone using the following equation:

$$\text{TI}=\frac{{\text{EC}}_{50}\;\text{for}\;\text{serum}\;{\text{K}}^{+}}{{\text{EC}}_{50}\;\text{for}\;\text{UACR}}$$ (1)

#### PK/pharmacodynamic (PD) Modeling Analysis for UACR and Serum K^+^

PK/PD modeling analysis for UACR and serum K^+^ was performed by FMD K&L (Fort Washington, PA, United States) using data for groups 2–5 of studies A and B. Sequential fits of PK and data were performed. KBP-5074 and eplerenone plasma concentrations were fitted to a one-compartment, first order absorption PK model with a single transit compartment and first order elimination, shown below. For eplerenone, the definition of k_tr_ = k_a_ was used, as k_tr_ derived a large k_a_ value (47.8). The Akaike information criterion (AIC), calculated for models with k_tr_ = k_a_ and k_tr_≠k_a,_ was 6,299 and 6,303, respectively. Hence, the k_tr_ = k_a_ model was used for the PD analysis of eplerenone. I_max_ was fixed to 1 to decrease the number of estimated parameters.

$$Depot\overset{k_{tr}}{\to }Abs.\overset{k_{a}}{\to }Central\overset{CL}{\to }$$ (2)

#### PK/PD Modeling Analysis for Urinary Na^+^/K^+^ Ratios

PK/PD modeling analysis was performed for urinary Na^+^/K^+^ ratios by Nuventra Inc. (Durham, NC, United States). Data from each of the five groups in studies A and B were used to evaluate the PK/PD relationship between urinary Na^+^/K^+^ ratios and average plasma concentrations of eplerenone or KBP-5074. Data from each study were analyzed independently according to a sigmoid E_max_ model using the equations below. The maximum effect of eplerenone or KBP-5074 on Na^+^/K^+^ ratio (E_max_) was calculated with the assumption that the maximum possible response was the Na^+^/K^+^ ratio from group 1 (Vehicle Sham group). Baseline (BSL) was defined as the Na^+^/K^+^ ratio for group 2 (Vehicle Aldosterone group) on Day 25 and was estimated by multiplying E_max_ by the effect of aldosterone (ALD). C_avg_ was defined as the average concentration of eplerenone or KBP-5074 on Day 25.

$$\begin{array}{l} Na^{+}/K^{+}\;\;Ratio=BSL+\frac{\left(Emax-BSL\right)\times C_{{\mathrm{avg}}^{\gamma }}}{EC_{50}^{\gamma }+C_{{\mathrm{avg}}^{\gamma }}}\\ BSL=E_{\max}\times ALD \end{array}$$ (3)

where γ = shape coefficient characterizing the sigmoidicity of the PK/PD relationship

$$C_{avg}=\frac{AUC_{\mathrm{last}}}{T_{last}}$$ (4)

#### Histology

Sections of paraffin-embedded kidney tissue 4 µm thick were stained to visualize collagen with Sirius Red and Masson’s Trichrome Stain. Sirius Red slides were analyzed by digital pathological analysis using HALO software from Indica Labs (Albuquerque, NM) to quantify the Sirius Red-positive area of the whole slide. Masson slides were read blindly and assigned an interstitial fibrosis score using a modification of the quantitative criteria from the Banff lesion grading system. Fibrosis of ≤5% of the cortical area was given a score of 0, 6–25% a score of 1, 26–50% a score of 2, 51–75% a score of 3, and >75% a score of 4 (Loupy et al., 2017).

#### Statistical Analyses

Body weight, urine volume, food and water intake, and biomarker values were expressed as means ± the standard error of the mean. Differences among and within groups were evaluated by two-way ANOVA Tukey’s multiple comparisons tests and the Sirius Red and Masson Trichrome Stain results were evaluated by unpaired t test using GraphPad Prism software v6.0 (Irvine, CA, United States). A difference was statistically significant when *p* < 0.05.

## Results

### Effect of KBP-5074 and Eplerenone on Body Weight, Food and Water Intake, and Urinary Output

There were no significant differences in baseline levels of body weight or food and water intake among any of the groups in either study. Body weight was significantly lower in the Vehicle Aldosterone group than in the Vehicle Sham group (*p* < 0.01 in Study A and *p* < 0.001 in Study B) at Day 27. Body weight was also significantly higher at Day 27 in the high-dose (450 mg/kg) eplerenone group and the mid- and high-dose KBP-5074 groups (1.5 and 5 mg/kg) than in the Vehicle Aldosterone group (*p* < 0.001, *p* < 0.0001, and *p* < 0.0001, respectively, Supplementary Figure S1). Improvement in physical condition, as determined by standard animal health evaluation, was observed in the groups with significant increases in body weight compared with those without. Urine volume was significantly higher in the Vehicle Aldosterone group than in the Vehicle Sham group on Days 7, 14, and 25 in both studies (*p* < 0.05 for all comparisons; Supplementary Figure S2). Compared with the Vehicle Aldosterone group, urine volume was significantly lower in the high-dose groups of both eplerenone and KBP-5074 at Days 7, 14, and 25, and in the KBP-5074 mid-dose group at Day 14 (*p* < 0.05 for all comparisons; Supplementary Figure S2). No dose of KBP-5074 significantly impacted food intake compared with the Vehicle Aldosterone group at any time during study B. In contrast, the administration of eplerenone 450 mg/kg significantly increased food intake from Day 15 to Day 23 (*p* < 0.05; Supplementary Figure S3). Water intake was significantly higher at all assessments in the Vehicle Aldosterone group than in the Vehicle Sham group in both studies (*p* < 0.001 for all comparisons). Compared with the Vehicle Aldosterone group, high-dose (450 mg/kg) eplerenone administration significantly decreased daily water intake from Day 1 to Day 4 and from Day 8 to Day 27 (*p* < 0.05 for both comparisons), high-dose (5 mg/kg) KBP-5074 administration significantly decreased daily water intake at all time points, and mid-dose (1.5 mg/kg) KBP-5074 significantly decreased daily water intake from Day 1 to Day 4 and from Day 8 to Day 12 (*p* < 0.05 for all comparisons; Supplementary Figure S3).

### Chronic Effects of Eplerenone and KBP-5074 on UACR and Serum K^+^

**FIGURE 1:**
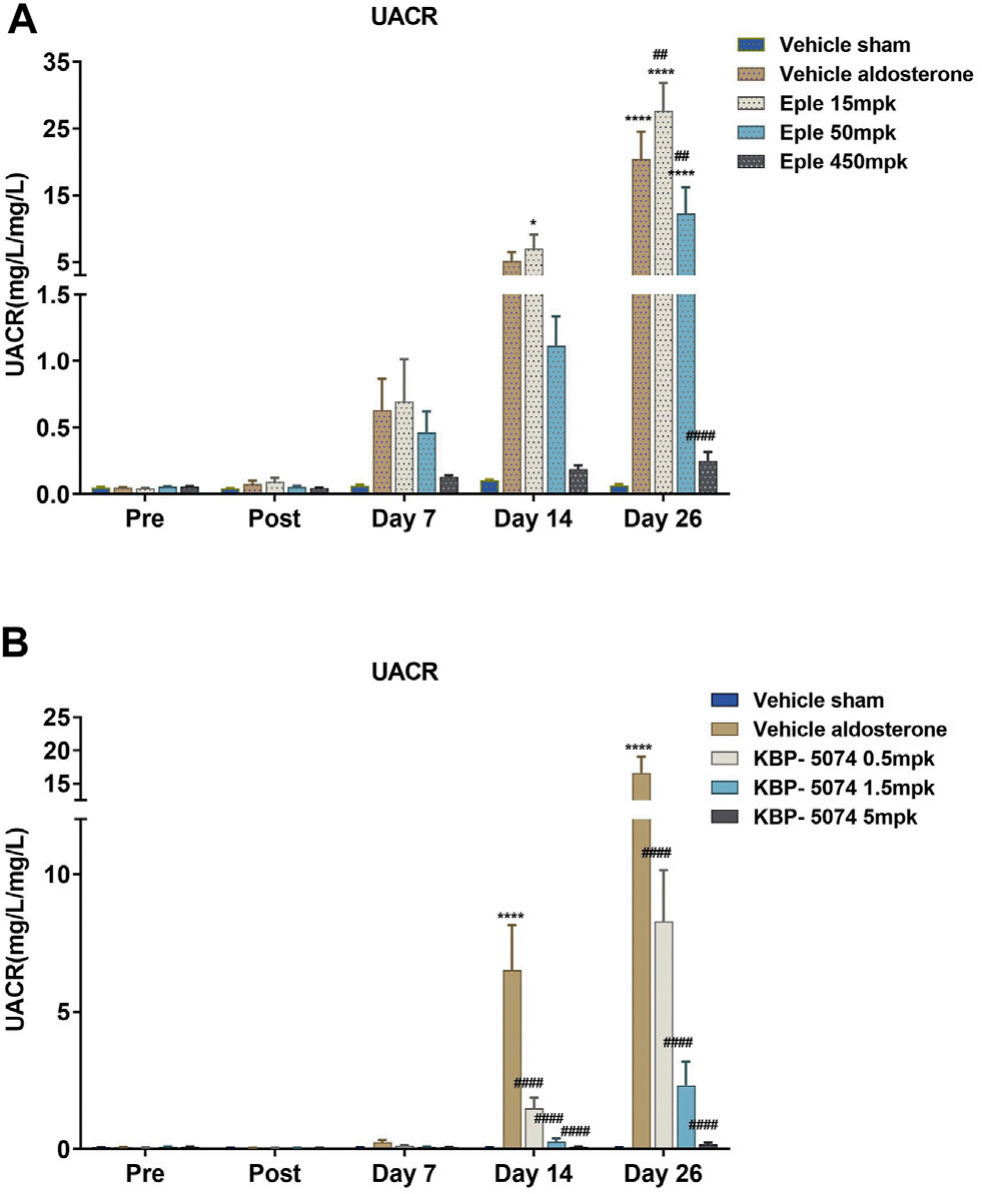
Effects of eplerenone and KBP-5074 on UACR. **(A)** Eplerenone: Values are expressed as mean ± SEM. *n* = 11–13. *p < 0.05, ****p < 0.0001 vs. Vehicle Sham group, ##*p* < 0.01, ####*p* < 0.0001 vs. Vehicle Aldosterone group followed by two-way ANOVA Tukey’s multiple comparisons test. **(B)** KBP-5074: Values are expressed as mean ± SEM. *n* = 11–13. ****p < 0.0001 vs. Vehicle Sham group, ####*p* < 0.0001 vs. Vehicle Aldosterone group followed by two-way ANOVA Tukey’s multiple comparisons test. Eple, eplerenone; SEM, standard error of the mean; UACR, urinary albumin-to-creatinine ratio.

No differences in baseline levels of UACR or serum K^+^ were observed among groups in either study (Figure 1). Significant increases in UACR were seen in the Vehicle Aldosterone group relative to the Vehicle Sham group in study B by Day 14 (*p* < 0.0001), and in both study A and B at Day 26 (*p* < 0.0001 for both). On Day 26, UACR was significantly lower in the eplerenone mid- and high-dose (50 and 450 mg/kg) groups than in the Vehicle Aldosterone group (*p* < 0.01 for both comparisons). UACR was significantly lower than in the Vehicle Aldosterone group in all KBP-5074 dose groups (0.5, 1.5, and 5 mg/kg) on Days 14 and 26 (*p* < 0.0001 for all comparisons). The effect on UACR of both eplerenone and KBP-5074 at Day 26 was found to be dose-dependent.

**FIGURE 2:**
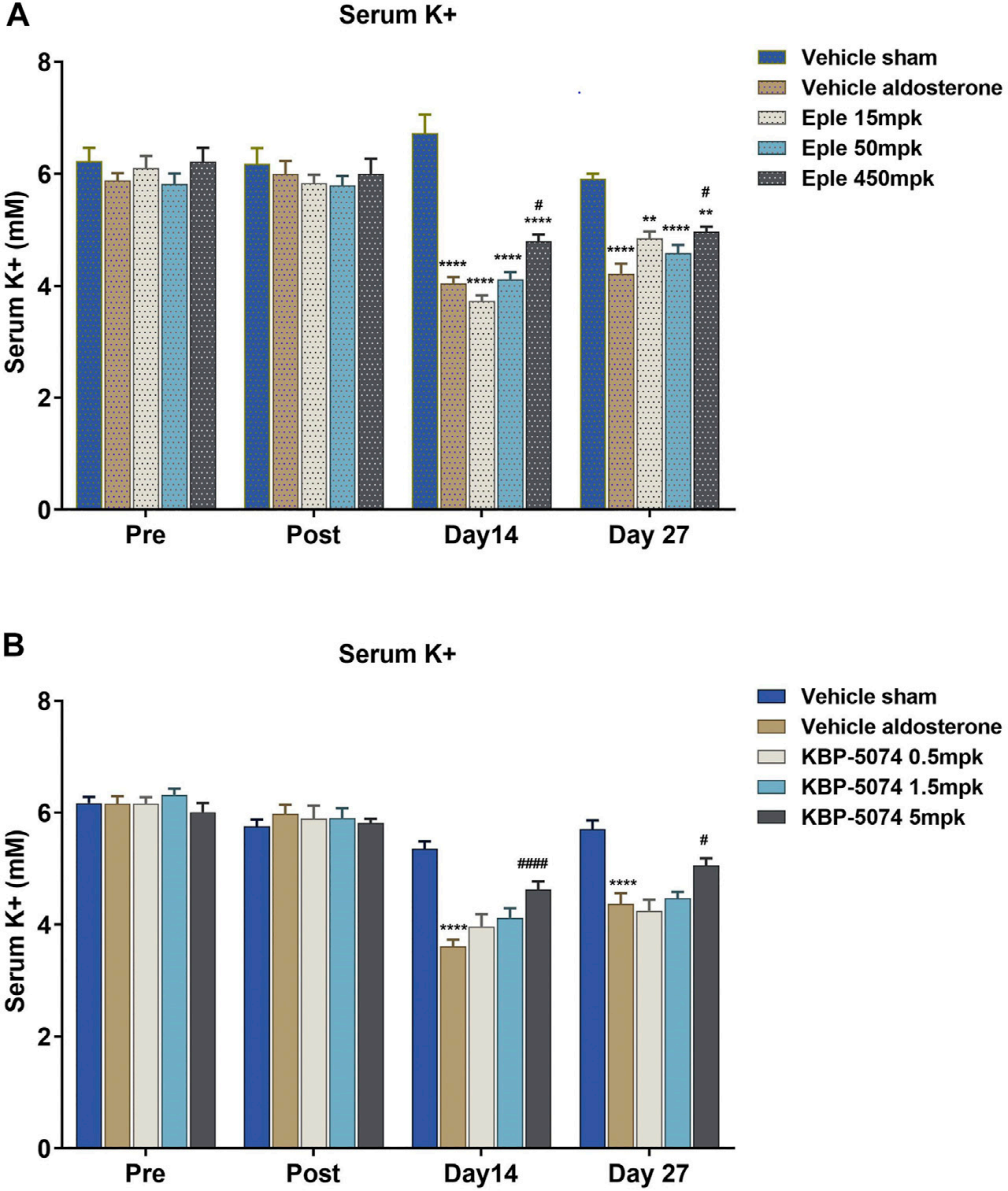
Effect of eplerenone and KBP-5074 on serum K^+^. **(A)** Eplerenone: Values are expressed as mean ± SEM. *n* = 11–13. **p < 0.01, ****p < 0.0001 vs. Vehicle Sham group, #*p* < 0.05 vs. Vehicle Aldosterone model group followed by two-way ANOVA Tukey’s multiple comparisons test. **(B)** KBP-5074: Values are expressed as mean ± SEM. *n* = 11–13. ****p < 0.0001 vs. Vehicle Sham group, #*p* < 0.05, ####*p* < 0.0001 vs. Vehicle Aldosterone group followed by two-way ANOVA Tukey’s multiple comparisons test. Eple, eplerenone; SEM, standard error of the mean.

Serum K^+^ was significantly lower in the Vehicle Aldosterone group than in the Vehicle Sham group by Day 14 in both studies (*p* < 0.0001 for both) (Figure 2). Significant increases in serum K^+^ levels were seen in both the high-dose eplerenone and high-dose KBP-5074 groups by Days 14 and Day 27 (*p* < 0.05 for all comparisons) compared with the Vehicle Aldosterone groups. Serum K^+^ increased in a dose-dependent manner for all dose groups of eplerenone and KBP-5074 at Day 14, but for KBP-5074 only at Day 27. The serum K^+^ did not reach or go above that of the Vehicle Sham group at any dose for either drug.

### Chronic Effects of Eplerenone and KBP-5074 on Urinary Na^+^, K^+^, and Na^+^/K^+^ Ratio

**FIGURE 3:**
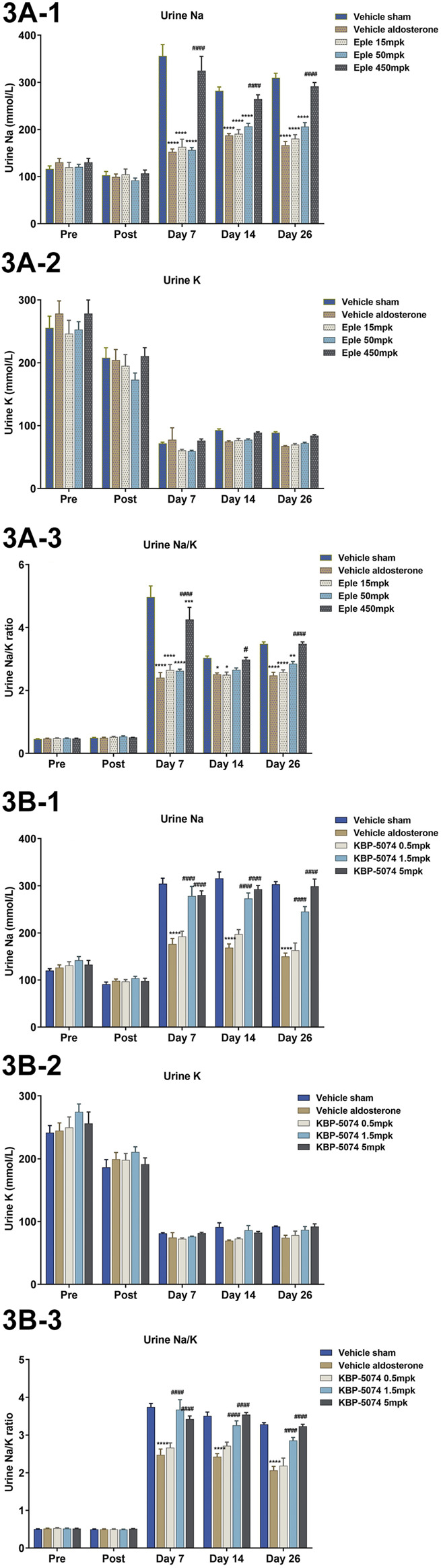
Effects of eplerenone and KBP-5074 on urine Na^+^, K^+^, and Na^+^/K^+^ ratio in **(A)** eplerenone and **(B)** KBP-5074. Values are expressed as mean ± SEM. *n* = 11–13. *p < 0.05, **p < 0.01, ****p < 0.0001 vs. Vehicle Sham group, #*p* < 0.05, ####*p* < 0.0001 vs. Vehicle Aldosterone group followed by two-way ANOVA Tukey’s multiple comparisons test. Eple, eplerenone; SEM, standard error of the mean.

In both study A and B, urinary Na^+^ was significantly lower in the Vehicle Aldosterone group than in the Vehicle Sham group at Days 7, 14 and 26 (*p* < 0.0001 for all comparisons) (Figure 3). Urinary K^+^ levels were similar at all time points; hence, the net result was a decrease in the urinary Na^+^/K^+^ ratio at Days 7, 14, and 26 in the Vehicle Aldosterone group (*p* < 0.05 for all comparisons). Significant increases in urinary Na^+^ levels in the high-dose (450 mg/kg) eplerenone group vs. the Vehicle Aldosterone group, together with no significant difference in urinary K^+^ levels between the two groups, also resulted in a significant increase in the Na^+^/K^+^ ratio at Days 7, 14, and 26 (*p* < 0.05 for all comparisons). Similar findings were also seen with the KBP-5074 high-dose group (5 mg/kg; *p* < 0.0001), and most importantly, in the mid-dose group (1.5 mg/kg; *p* < 0.0001), even without significant differences in food/salt intake.

### PK of Eplerenone and KBP-5074

Plasma concentrations for eplerenone and KBP-5074 were determined for all three dose groups on Day 1 and Day 26 at 1, 2, 4, and 7 h post first dose, except for the 4-h sample for one rat in the eplerenone study, which was inadvertently missed. The C_max_ and AUC_0-last_ were calculated for eplerenone and KBP-5074 and are shown in Table 1. The data show a positive correlation between exposure and dose *in vivo* for both eplerenone and KBP-5074, with values increasing from the low to high doses and between Day 1 and Day 26.

**TABLE 1:** PK parameters of eplerenone and KBP-5074 in plasma on Day 1 and Day 26.

Compound	Concentration	Day 1		Day 26	
		C_max_ (ng/ml)	AUC_0-last_ (ng h/ml)	C_max_ (ng/ml)	AUC_0-last_ (ng h/ml)
Eplerenone	15 mg/kg	1,049 ± 281	1875 ± 672	1,179 ± 817	1939 ± 1,683
	50 mg/kg	3,629 ± 1,375	9,100 ± 2,488	3,486 ± 1,302	4,912 ± 1,573
	450 mg/kg	24,285 ± 7,084	107,179 ± 22,230	10,355 ± 3,303	24,097 ± 6,357
KBP-5074	0.5 mg/kg	50.5 ± 13.1	258.0 ± 66.3	83.1 ± 29.3	378.0 ± 111.0
	1.5 mg/kg	171.0 ± 33.2	834.0 ± 143.0	273.0 ± 71.6	1,505.0 ± 376.0
	5.0 mg/kg	568.0 ± 158.0	2,806.0 ± 693.0	834.0 ± 266.0	5,166.0 ± 1,805.0

Data expressed as mean ± standard error of the mean.

AUC_0–last_, area under the concentration time–curve from zero to last measurement; C_max_, maximum serum concentrations; PK, pharmacokinetics.

The PK parameters calculated in the PK/PD modeling analysis are shown in Table 2. Clearance (CL) estimates were 6.17 and 0.734 L/h with volume (V) estimates of 0.034 L and 11.6 L for eplerenone and KBP-5074, respectively. All percent relative standard errors (%RSE) were less than 50%, with the exception of the %RSE of V for eplerenone, which was 68.9%.

**TABLE 2:** PK/PD modeling analysis: PK parameters for eplerenone and KBP-5074.

	Parameter	Estimate	%RSE	BSV (CV%)	Shrink (%)
Eplerenone	CL (L/h)	6.17	3.61	29.4	20.0
	V (h)	0.034	68.9	102.0	93.2
KBP-5074	CL (L/h)	0.734	32.5	65.5	2.93
	V (L)	11.6	3.51	41.2	16.0

BSV, between-subject variability; CL, clearance; %RSE, percent relative standard errors; PD, pharmacodynamics; PK, pharmacokinetics; V, volume.

### PK/PD Modeling of UACR and Serum K^+^

The PD parameters for UACR and serum K^+^ were calculated and are shown in Table 3. The %RSEs of EC_50_ were all less than 50%. Modeling analysis results are shown in Table 4. EC_50_ values for serum K^+^ were 666 nM (95% CI 53.6–8,275) and 538 nM (95% CI 106–2,738) for eplerenone and KBP-5074, respectively. EC_50_ values for UACR were 1,071 nM (95% CI 384–2,968) and 22.0 nM (95% CI 5.77–84.3) for eplerenone and KBP-5074, respectively. The TI of KBP-5074 was 24.5 compared with a TI of 0.620 for eplerenone.

**TABLE 3:** PK/PD modeling analysis: PD parameters for eplerenone and KBP-5074 for serum K^+^ and UACR. (A) Serum K^+^

	Parameter	Estimate	%RSE	BSV (CV%)	Shrink (%)
Eplerenone	E_max_	0.151	13.5	18.5	84.5
	EC_50_ (ng/ml)	276.0	22.9	48.7	87.8
	K_out_ (/h)	0.0879	16.7	132.0	90.1
	K_in_ (mmol/L/h)	3.95	1.74	0.916	87.8
	cov	0.11	6.97	33.8	68.0
KBP-5074	E_max_	0.318	24.7	9.14	89.0
	EC_50_ (ng/ml)	271.0	14.8	51.1	78.3
	K_out_ (/h)	0.0130	5.18	4.66	54.2
	K_in_ (mmol/L/h)	0.0489	6.93	2.19	76.9
	cov	0.0864	13.1	65.5	58.9

	Parameter	Estimate	%RSE	BSV (CV%)	Shrink (%)
(B) UACR				
Eplerenone	E_max_	0	FIXED	5.60	85.8
	EC_50_ (ng/ml)	444.0	8.57	39.1	83.1
	K_out_ (/h)	0.000114	62.6	16.3	84.1
	K_in_ (mmol/L/h)	112.0	119.0	132.0	9.42
KBP-5074	E_max_	0	FIXED	NA	NA
	EC_50_ (ng/ml)	11.1	28.4	541.0	51.1
	K_out_ (/h)	4.65e-6	905.0	187.0	95.4
	E_0_	0.202	5.33	61.0	87.8

BSV, between-subject variability; CL, clearance; E_max_, maximum possible response; EC_50_, concentration of drug producing 50% of maximum effect; K_out_, rate of elimination; K_in_, rate of infusion; %RSE, percent relative standard errors; PD, pharmacodynamics; PK, pharmacokinetics.

**TABLE 4:** Effect of eplerenone and KBP-5074 on UACR and serum K^+^.

Compound	Endpoint	EC_50_ (nM)	95% CI lower limit	95% CI upper limit	TI	KBP-5074/Eplerenone TI ratio
KBP-5074	Serum K^+^	538	106.0	2,738	24.500	39.5
	UACR	22.00	5.77	84.30		

Eplerenone	Serum K^+^	666	53.6	8,275	0.620	
	UACR	1074.00	384.00	2968.00		

Data expressed as EC_50_ with 95% CI; the TI was calculated as the EC_50_ for serum K^+^/EC_50_ for the UACR. CI, confidence interval; EC_50_ concentration of drug producing 50% of maximum effect; TI, therapeutic index; UACR, urine albumin-to-creatinine ratio.

### PK/PD Modeling of Urinary NA^+^/K^+^ Ratio

**FIGURE 4:**
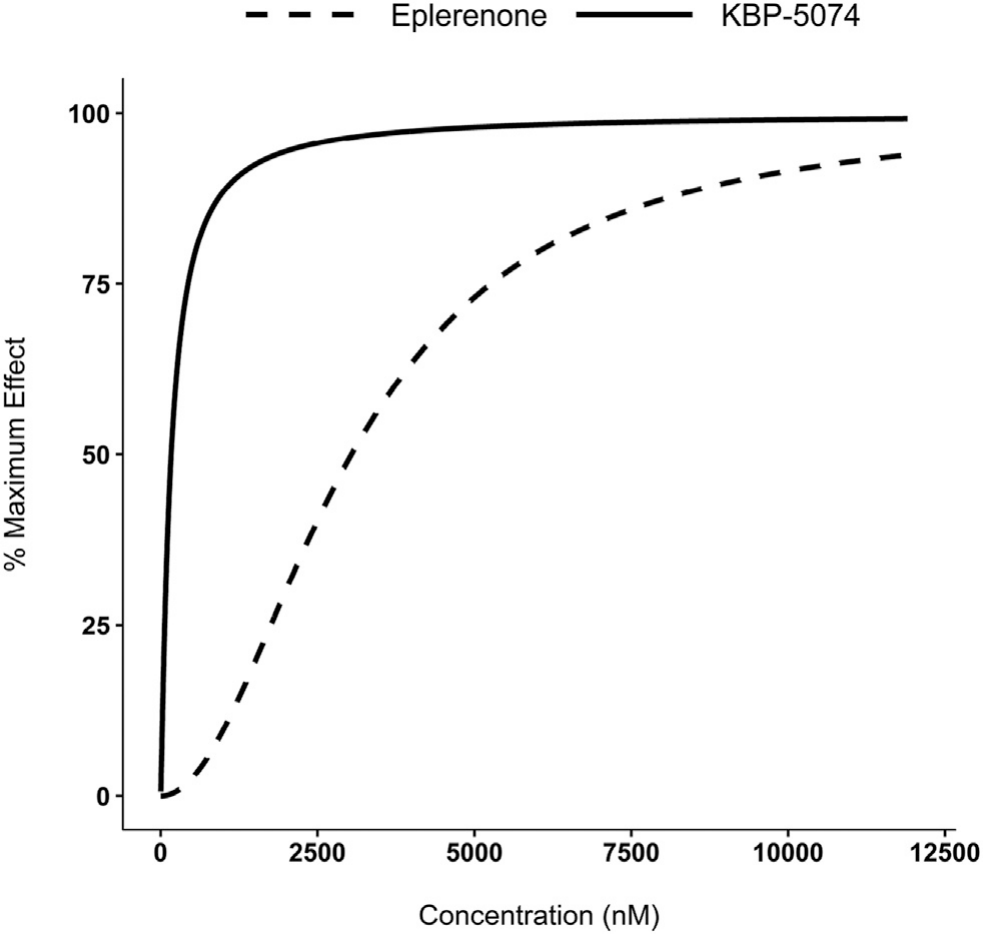
PK/PD modeling of urinary Na^+^/K^+^ ratios. Concentration-effect curves of simulated Na^+^/K^+^ ratios as a function of eplerenone and KBP-5074 concentrations. The Na^+^/K^+^ ratio data were normalized to range from 0% (baseline response) to 100% (maximum response).

Results are shown in Table 5. The EC_50_ values were estimated to be 3,040 nM and 167 nM for eplerenone and KBP-5074, respectively, indicating that KBP-5074 is approximately 18-fold more potent than eplerenone at increasing the Na^+^/K^+^ ratio based on plasma concentrations. Figure 4 shows a comparison of the simulated Na^+^/K^+^ ratio as a function of either KBP-5074 or eplerenone concentration. The γ estimate was approximately 1 for KBP-5074, showing that the Na^+^/K^+^ ratio increased approximately linearly prior to a final asymptotic approach to E_max_. In contrast, the γ estimate was approximately 2 for eplerenone, consistent with an initial, slower rise in the Na^+^/K^+^ ratio as a function of plasma concentration. Based on this modeling, KBP-5074 has a steeper exposure response compared with eplerenone.

**TABLE 5:** PK/PD parameter estimates for urinary Na^+^/K^+^ ratio for eplerenone and KBP-5074.

Parameter	Estimate (%RSE)	
	Eplerenone	KBP-5074
ALD	0.72 (3.8%)	0.63 (5.0%)
EC_50_ (nM)	3,040 (29.5%)	167 (36.9%)
E_max_ ^a^	3.54 (1.5%)	3.31 (1.3%)
γ	2.00 (33.5%)	1.14 (23.3%)

a Maximum Na^+^/K^+^ response (E_max_) was assumed to be equivalent to the response in group 1, the Vehicle Sham group; ALD, aldosterone; E_max_, maximum possible response; EC_50_, concentration of drug producing 50% of maximum effect; %RSE, percent relative standard error of the parameter estimate; PD, pharmacodynamics; PK, pharmacokinetics; γ, shape coefficient characterizing the sigmoidicity of the PK/PD relationship.

### Effect of Eplerenone and KBP-5074 on Collagen

Collagen staining was performed for the Vehicle Aldosterone and high-dose groups (450 mg/kg and 5 mg/kg for eplerenone and KBP-5074, respectively) of each study using Sirius Red and Masson’s Trichrome Stain. Representative images and results are shown in Supplementary Figure S4. Average Sirius Red-positive staining on the slide was 15.02 vs. 9.18% (*p* < 0.001) and 14.96 vs. 9.44% (*p* < 0.1) for the Vehicle Aldosterone and high-dose groups of study A and study B, respectively. For the Masson staining, the average interstitial fibrosis Banff lesion grade score was 1.77 vs. 0.31 (*p* < 0.0001) and 2.62 vs. 0.54 (*p* < 0.0001) for the Vehicle Aldosterone and high-dose groups of study A and study B, respectively.

## Discussion

We compared the novel non-steroidal MRA KBP-5074 with the steroidal MRA eplerenone in parallel studies using a rat model of mineralocorticoid-induced renal injury. TI was calculated as the ratio of the EC_50_ for increasing serum K^+^ to the EC_50_ of decreasing UACR and was found to be greater for KBP-5074 than for eplerenone. Based on these findings, KBP-5074 merits development for the treatment of nephropathy in patients with high risk of hyperkalemia.

Aldosterone binds to the MR in the epithelium of the distal nephron and modulates both sodium reabsorption and potassium secretion. Remnant rat models have shown that the addition of aldosterone increases proteinuria and glomerulosclerosis in the kidney, even reversing the protection seen with ACEI/ARB treatment (Greene et al., 1996). Steroidal MRAs, such as eplerenone and spironolactone, have been shown to decrease proteinuria in CKD patients and have long been used for the management of hypertension and HF, but have safety concerns due to the risk of hyperkalemia (Alexandrou et al., 2019; Barrera-Chimal et al., 2019). The aldosterone-salt model used for this study, as well as the similar DOCA-salt model, have been used historically to test the potential benefits of some MRAs, including non-steroidal MRAs such as esaxerenone and BR-4628, a precursor to finerenone (Schupp et al., 2011; Arai et al., 2016).

In the current study, we compared the TIs, efficacy, and natriuretic impact of the novel non-steroidal MRA KBP-5074 with the steroidal MRA eplerenone in the uninephrectomized SD rat model of aldosterone/salt-induced renal damage. Aldosterone/salt treatment of uninephrectomized rats increased UACR, decreased serum K^+^, and decreased urinary Na^+^ compared with the Vehicle Sham control group, as expected, validating the model of aldosterone/salt-induced renal damage used to perform the study. While treatment with both MRAs reduced these effects, KBP-5074 showed increased efficacy compared with the steroidal MRA eplerenone, as measured by UACR, preventing the increase seen in the Vehicle Aldosterone control group in a dose-dependent manner at all doses tested; in contrast, eplerenone was effective only in the mid- and high-dose groups. Both KBP-5074 and eplerenone showed similar effects on serum K^+^, blunting the hypokalemia induced by the aldosterone challenge in the Vehicle Aldosterone group with significant increases noted only in the high-dose groups for each. The histology data is supportive of these findings, showing a significant decrease in interstitial fibrosis as measured by collagen staining with eplerenone and KBP-5074 at the highest concentrations when compared with the Vehicle Aldosterone group. There was a substantial increase in urinary Na^+^ excretion with KBP-5074 in the mid- and high-dose groups but only at the high concentration of eplerenone, which is consistent with the significant increase in food/salt intake found in this group. PK/PD modeling of urinary Na^+^/K^+^ ratio showed that KBP-5074 has a steeper exposure response. The UACR and serum K^+^ results are consistent with a previous study comparing another non-steroidal MRA PF-03882845 with eplerenone, although it did not report a significant increase in urinary Na^+^ at any dose of eplerenone (Orena et al., 2013). Further, PK/PD analysis showed that KBP-5074 was more effective at reducing UACR without significant increase of potassium, as evidenced by the TI.

KBP-5074 demonstrated a 39-fold increase in TI compared with eplerenone. This difference is driven primarily by greater reduction of UACR in the KBP-5074 treatment groups as both KBP-5074 and eplerenone showed similar effects on serum K^+^. The observed difference in reduction of UACR between KBP-5074 and eplerenone may be due to several factors relating to the differences between non-steroidal and steroidal MRAs.

Two recent studies in humans support the preclinical findings from the aldosterone-salt and DOCA-salt rat models in studies of esaxerenone and the finerenone precursor, mentioned above, validating translation of findings from these animal models to humans. A phase three, randomized, controlled, clinical trial of esaxerenone in patients with type 2 diabetes and microalbuminuria found that its addition to existing RAAS inhibitor therapy significantly improved albuminuria levels more quickly and slowed the progression of albuminuria increase with only asymptomatic increases in serum K^+^ levels that were identified and resolved with dosage adjustments or discontinuation of treatment (Ito et al., 2019). In a double-blind trial of chronic kidney disease outcomes in patients with type 2 diabetes, finerenone treatment resulted in lower risks of CKD progression and cardiovascular events, with 2.3% of patients requiring discontinuation of therapy due to hyperkalemia-related events vs. 0.9% in the control group (Bakris et al., 2020). Recent studies have strengthened the rationale for the use of serum K^+^ as a marker of safety. When serial K^+^ measurements were integrated into a risk model for the initiation and maintenance of MRA treatment, risk prediction of cardiovascular death in patients with HF eligible for RAAS inhibitors and MRA therapy was improved. Patients with abnormal K^+^ displayed poorer outcomes, independent of history, other clinical and laboratory parameters, and treatment parameters. (Rossignol et al., 2020).

In daily practice, many patients receive suboptimal doses of RAAS inhibitors or discontinue the use of RAAS inhibitors or MRAs even though there is clear evidence to support their benefit, mainly due to concerns about worsening renal function and hyperkalemia (Vijayakumar et al., 2019). This is clinically important, as MRA therapy is a critical component of disease management programs (Rossignol et al., 2019). One study found that in the first year after MRA initiation, 18% of patients experienced at least one episode of hyperkalemia, a proportion three times higher than that observed for matched individuals starting beta-blockers. The hyperkalemia in MRA users occurred primarily within the first 3 months and resulted in discontinuation in 47% of cases, with CKD patients being at the highest risk for MRA discontinuation (Trevisan et al., 2018). These studies highlight the need for MRAs with a better TI regarding the risk of hyperkalemia, as well as careful investigation of the cause of hyperkalemia before discontinuation of MRAs.

Even though our results clearly show that KBP-5074 demonstrated an improved TI and increased efficacy compared with eplerenone, as well as evidence of reduction in aldosterone-induced hypokalemia and interstitial fibrosis, there are limitations to the study. Due to the large number of animals required for each study, it was not logistically possible to perform a single randomized study in parallel instead of two separate studies, but the overall amplitude of the differences between the Vehicle Sham and Vehicle Aldosterone control groups from the two studies were similar. While there were some differences in the intensity of the impact of the aldosterone treatment in the Vehicle Aldosterone groups between the studies, the outcomes themselves were similar, with increased UACR, decreased serum K^+^, and decreased urinary Na^+^ compared with the Vehicle Sham control group, allowing for comparison of the results. Additionally, the aldosterone-salt model does not take account of the possible activation of MR by glucocorticoids and the interactions between MRs and glucocorticoid receptors, and thus addresses only part of the pathophysiological effects (Gomez-Sanchez, 2019). The translatability of serum K^+^ studies from rodents to human is not well defined and the aldosterone rodent model is not an exact representation of the slower progression of disease seen in the CKD patient population due to the abbreviated timeline and combination of hyperaldosteronemia and high sodium load. Further studies measuring blood pressure, gene expression of collagen and other proinflammatory and profibrotic genes, examining additional histology, and showing reproducibility in another proteinuric CKD model are needed to confirm and expand upon these findings. Further, studying the effects of the optimal dose of KBP-5074 after renal injury has already developed would be critical for evaluating its use as a therapeutic option now that the initial proof-of-concept study has been completed. While the objective of this study was to compare KBP-5074 to the classical steroidal MRA eplerenone, comparisons to other non-steroidal MRAs, including, but not limited to, esaxerenone and finerenone, would also be of interest.

In conclusion, this study shows the advantage and superiority of the novel non-steroidal MRA KBP-5074 over eplerenone. Despite two separate studies having been performed to test the effects of eplerenone or KBP-5074 in the aldosterone-induced renal damage rodent model of nephropathy, the effect of aldosterone is consistent between both studies. KBP-5074 has been shown to limit albuminuria, with both improved efficacy and therapeutic index (TI) compared with eplerenone. Using urinary Na^+^ as an indirect marker, it also has the potential for blood pressure reduction. These findings merit further investigation of KBP-5074 in humans as an efficacious and safe treatment for CKD.

## Data Availability

The raw data supporting the conclusions of this article will be made available by the authors without undue reservation.
