# Supplementary material for: The Non-Steroidal Mineralocorticoid Receptor Antagonist KBP-5074 Limits Albuminuria and has Improved Therapeutic Index Compared With Eplerenone in a Rat Model With Mineralocorticoid-Induced Renal Injury
Source: Front Pharmacol. 2021 Jun 24;12:604928. doi: 10.3389/fphar.2021.604928 (PMC8264204; doi:10.3389/fphar.2021.604928)
Supplement: Supplementary file 1 [file DataSheet1.docx]

SUPPLEMENTARY FIGURES

Supplementary Figure 1. Effect of eplerenone and KBP-5074 on body weight.

1. Eplerenone


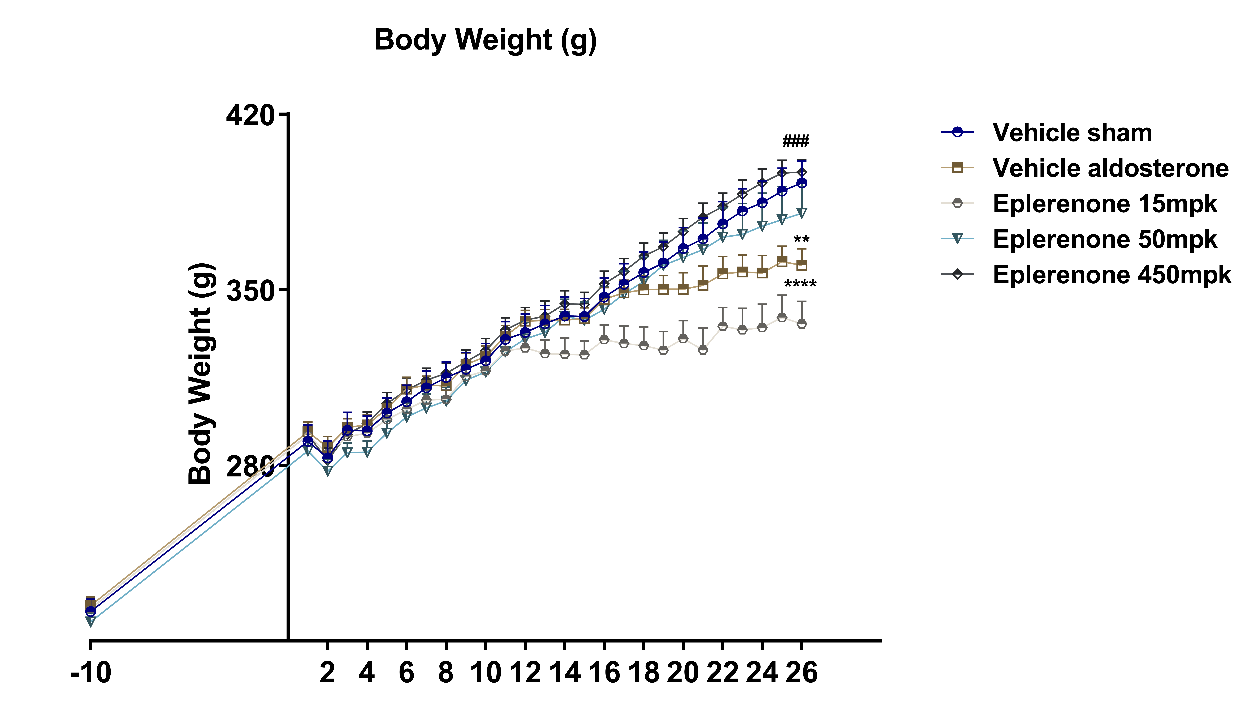


1. KBP-5074


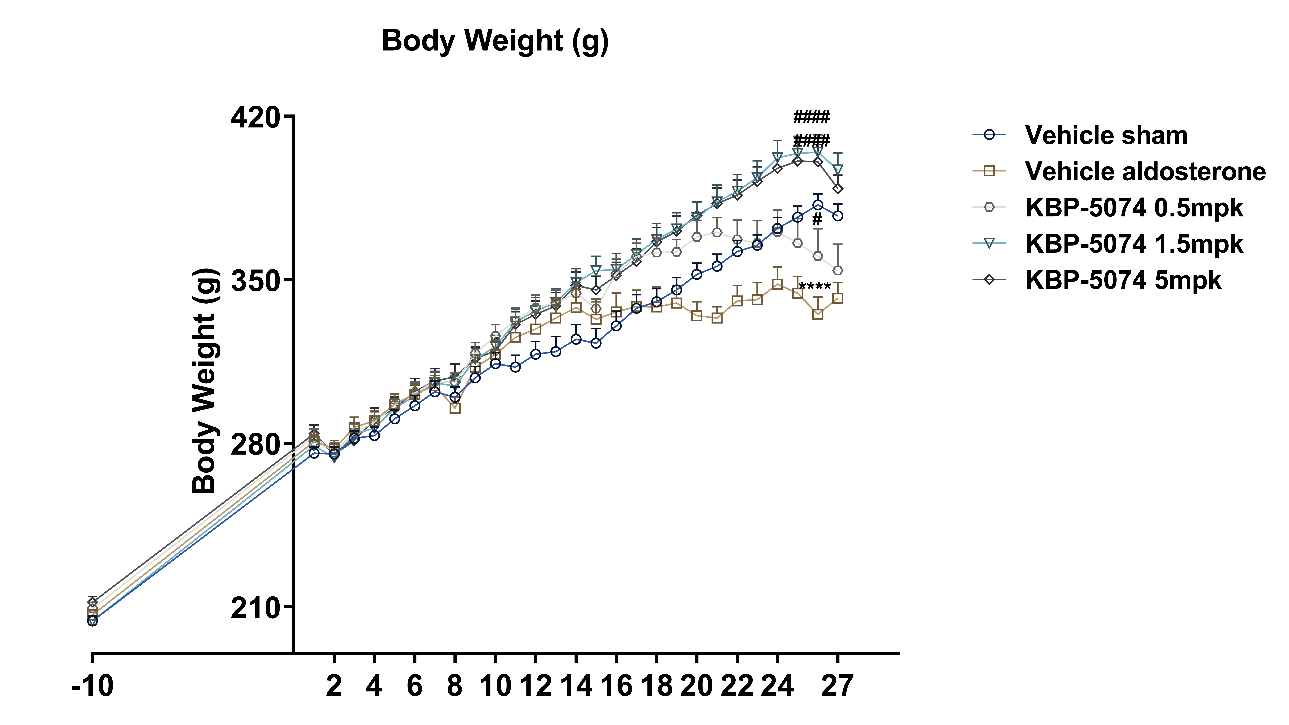


Values are expressed as mean±SEM. n=11–13. *P<0.05, ***P<0.001, ****P<0.0001 vs Vehicle Sham group, #P<0.05, ##P<0.01, ###P<0.001, ####P<0.0001 vs Vehicle Aldosterone group followed by two-way ANOVA Tukey’s multiple comparisons test.

Supplementary Figure 2. Effect of eplerenone and KBP-5074 on urine volume.

1. Eplerenone


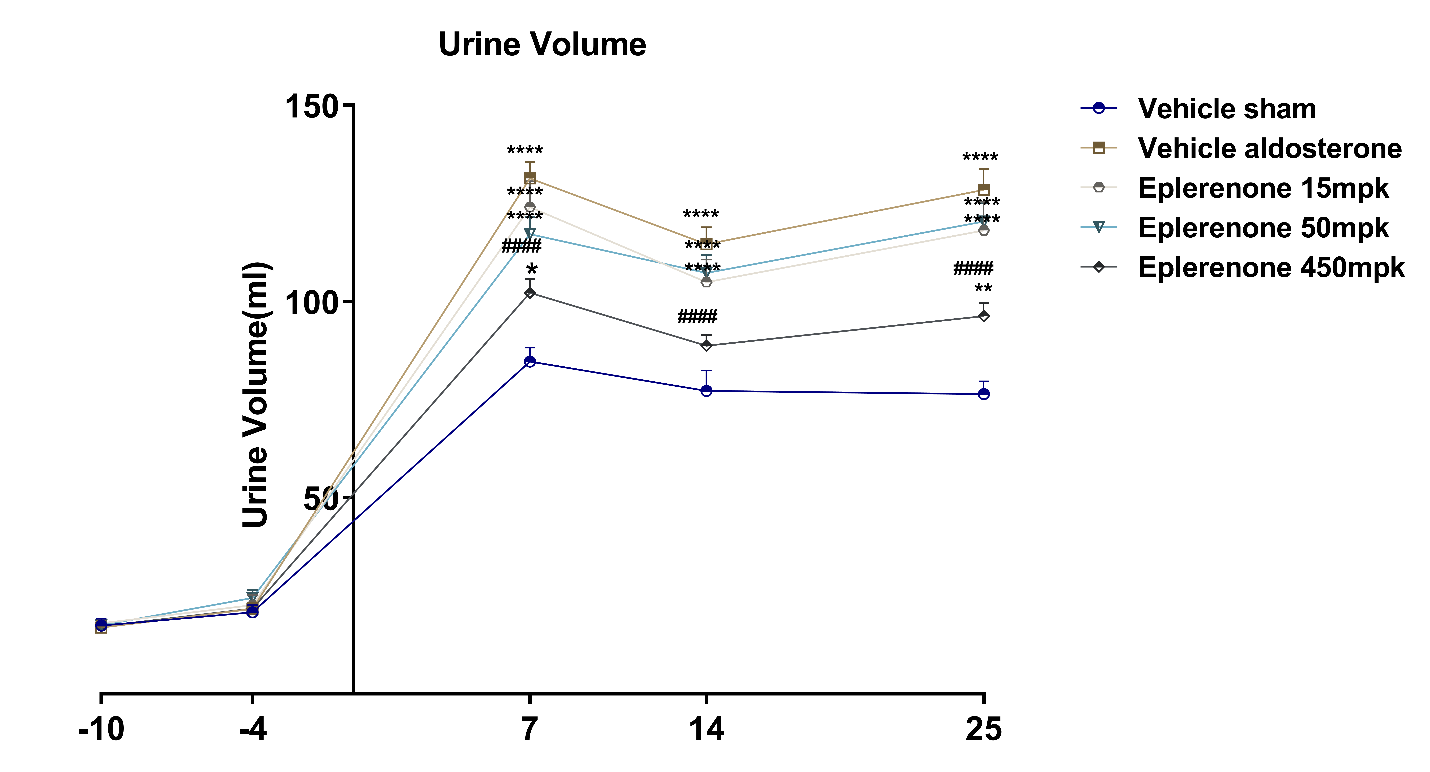


1. KBP-5074


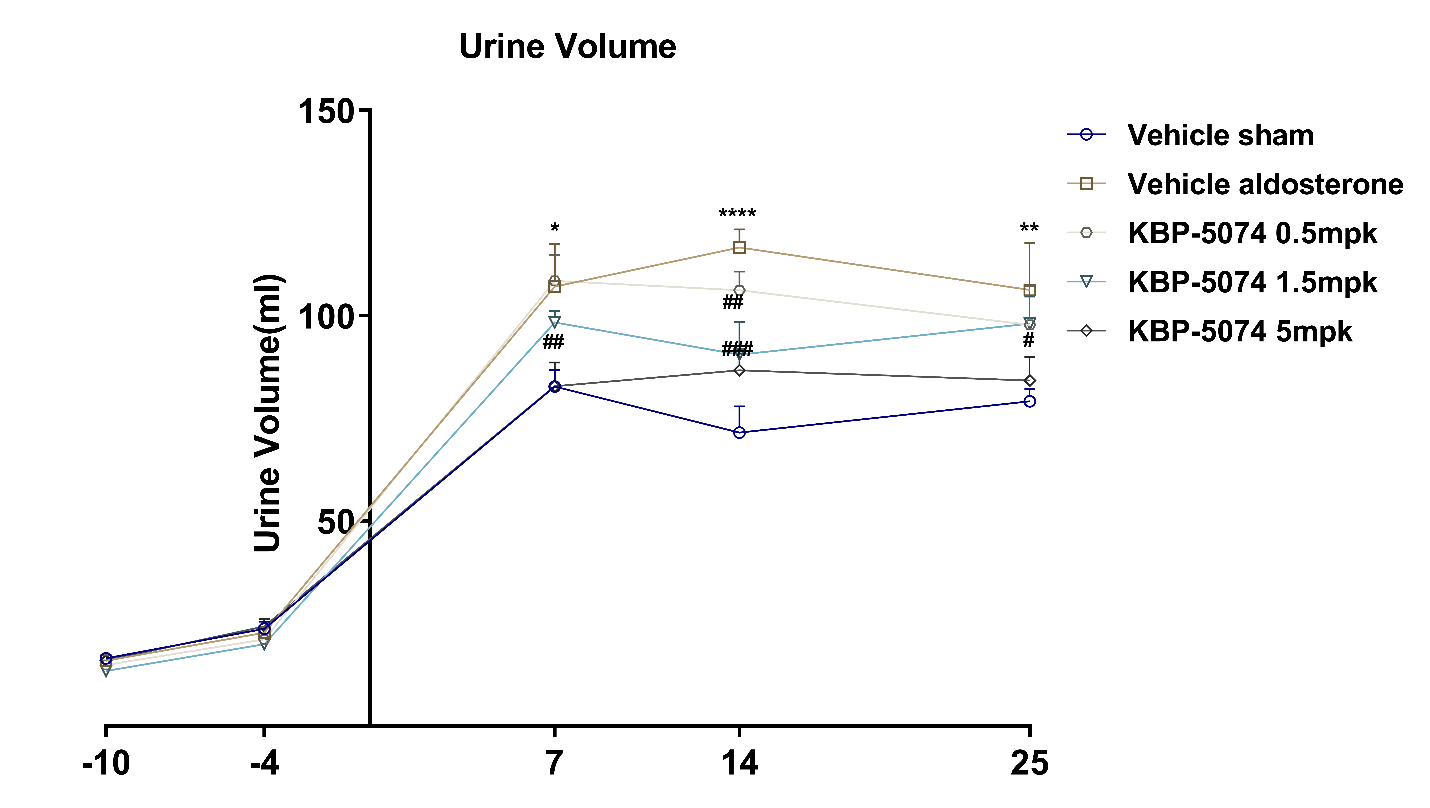


Values are expressed as mean±SEM. n=11–13. *P<0.05, ***P<0.001, ****P<0.0001 vs Vehicle Sham group, #P<0.05, ##P<0.01, ###P<0.001 ####P<0.0001 vs Vehicle Aldosterone group followed by two-way ANOVA Tukey’s multiple comparisons test.

Supplementary Figure 3. Effect of eplerenone and KBP-5074 on food and water intake.

1. Eplerenone food intake (B) Eplerenone water intake


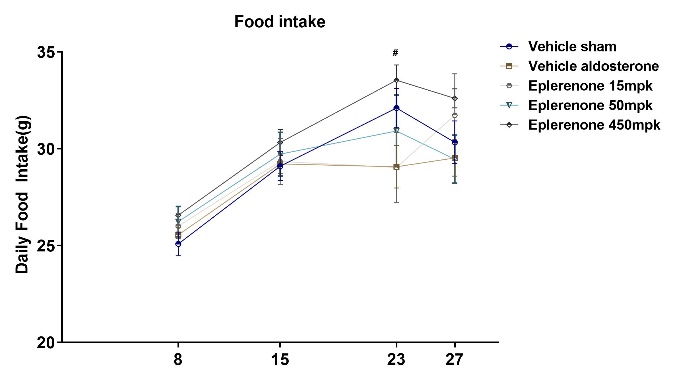

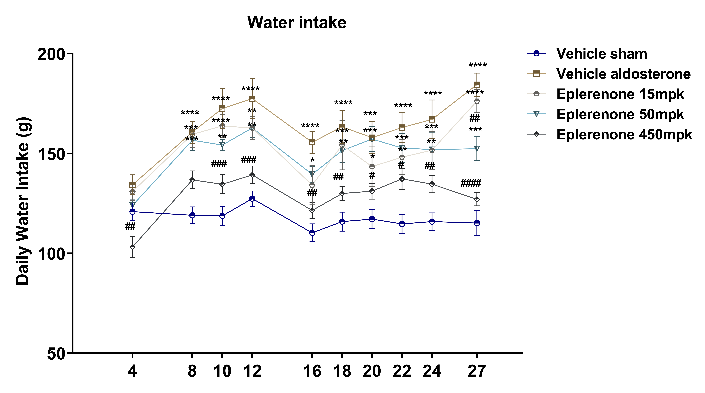


1. KBP-5074 food intake (D) KBP-5074 water intake


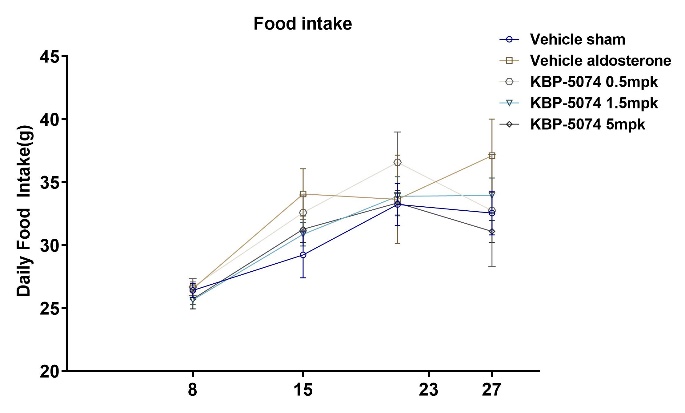

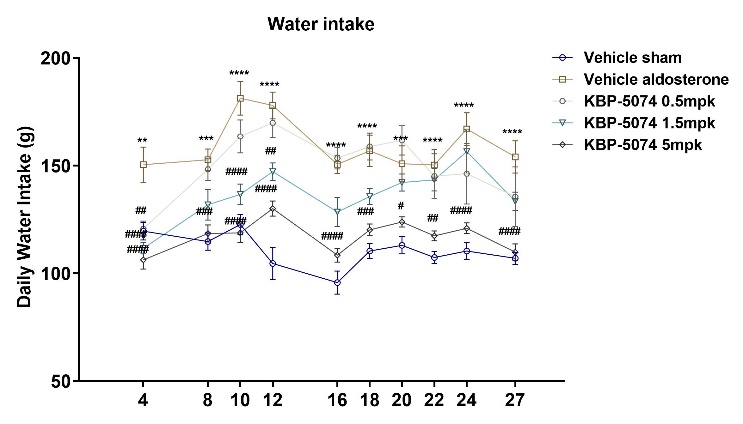


Values are expressed as mean±SEM. n=11–13. ***P*<0.01, ****P*<0.001, *****P*<0.0001 vs Vehicle Sham group, #*P*<0.05, ##*P*<0.01, ###*P*<0.001 ####*P*<0.0001 vs Vehicle Aldosterone group followed by two-way ANOVA Tukey’s multiple comparisons test.

Supplementary Figure 4. Effect of eplerenone and KBP-5074 on collagen immunohistochemical localization in the kidney.


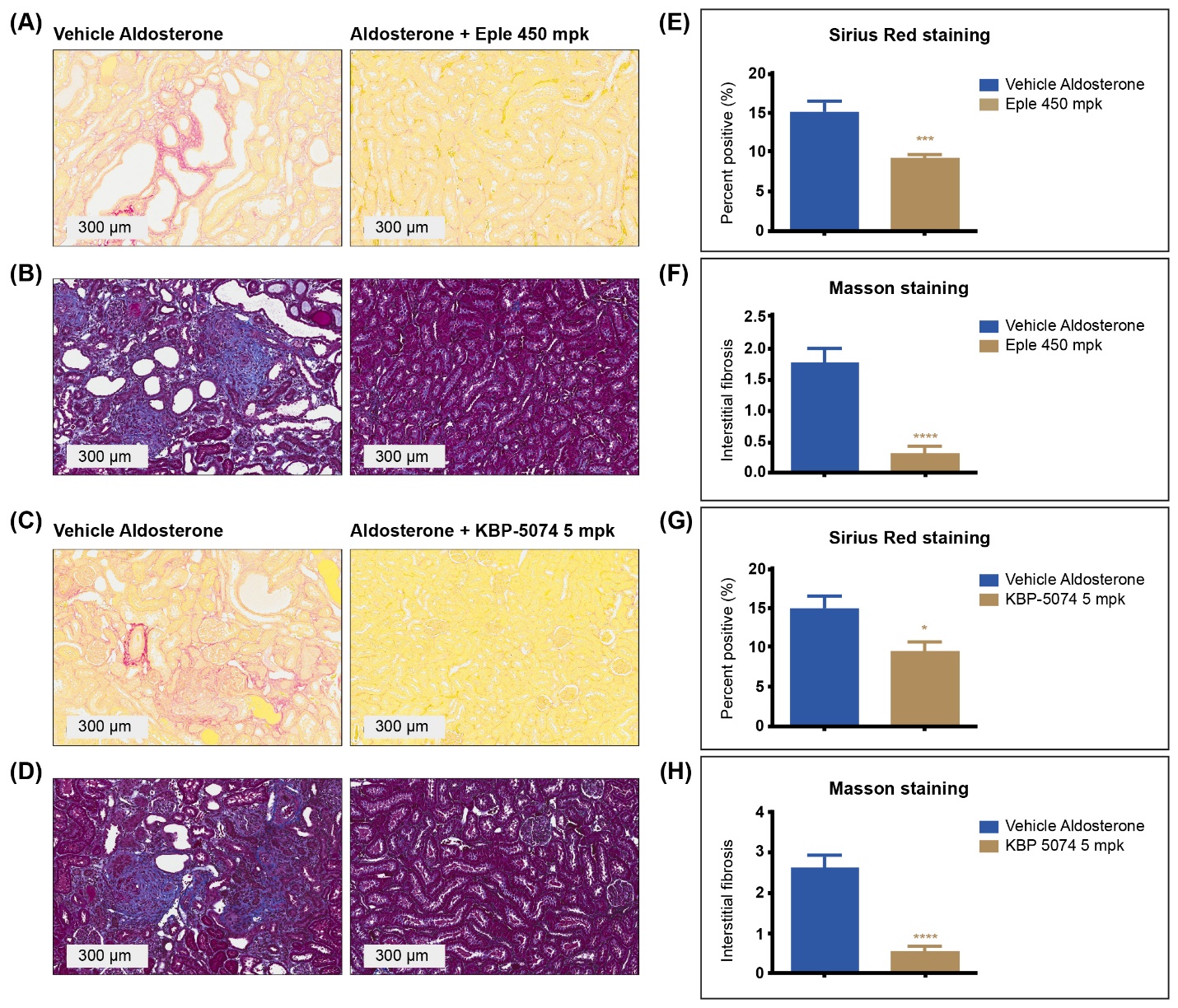


Representative images: White bar shows 300 µm scale (A) Study A, Vehicle Aldosterone group (left) and high-dose eplerenone (450 mg/kg) group (right), Sirius Red, (B) Study A, Vehicle Aldosterone group (left) and high-dose eplerenone (450 mg/kg) group (right), Masson’s Trichrome Stain, (C) Study B, Vehicle Aldosterone group (left) and high-dose KBP-5074 (5 mg/kg) group (right), Sirius Red, (D) Study B, Vehicle Aldosterone group (left) and high-dose KBP-5074 (5 mg/kg) group (right), Masson’s Trichrome Stain. Data expressed as % Sirius Red positive or Interstitial Fibrosis score (Masson) and represented as the mean±SEM, n=11-13: (E) and (F) Study A, (G) and (H) Study B. **P*<0.1, *** *P*<0.001, *****P*<0.0001.
